# Effect of ultrasonic surface deep rolling combined with oxygen boost diffusion treatment on fatigue properties of pure titanium

**DOI:** 10.1038/s41598-021-97255-x

**Published:** 2021-09-08

**Authors:** Xue-Fei Teng, Yun-Fei Jia, Cong-Yang Gong, Cheng-Cheng Zhang, Xian-Cheng Zhang, Shan-Tung Tu

**Affiliations:** 1 Key Laboratory of Pressure Systems and Safety, Ministry of Education, School of Mechanical and Power Engineering, East China University of Science and Technology, Shanghai, 200237 China; 2 Shanghai Engineering Research Center for Commercial Aircraft Engine, AECC Commercial Aircraft Engine Co. LTD, Shanghai, 201108 China

**Keywords:** Mechanical engineering, Structural materials

## Abstract

Ultrasonic surface deep rolling (USDR), oxygen boost diffusion (OBD), and their combination (USDR-OBD) were all used to improve the surface hardening of pure titanium. The microstructure, microhardness, and fatigue life of pure titanium treated by USDR, OBD, and USDR-OBD methods were analyzed. USDR treatment induced a severe deformation area, while OBD treatment produced a brittle oxygen diffusion zone. The USDR-OBD treated samples approached the highest hardness in comparison with other treated samples. The fatigue lives of USDR treated samples were improved, which was due to the high compressive residual stress and refined grains. However, the fatigue lives of both OBD treated samples and USDR-OBD treated samples were decreased due to premature crack initiation and rapid propagation in the oxygen diffusion zone. Finally, the fatigue fracture mechanisms of different samples were proposed.

## Introduction

Titanium and its alloys have the advantages of low density, good corrosion resistance, and excellent machinability^1^. However, because of the poor fatigue resistance and tribological properties, the application of titanium is restricted^1^. Various surface modification methods have been applied to enhance the surface properties and fatigue resistance of titanium and its alloys^2^. Among the existing methods, surface thermo-chemical methods, including nitriding^3^, carburizing^4^, boriding^5^, and oxidizing^6,7^, have been developed for improving the surface hardness and wear resistance. To get better surface properties, a technique for obtaining higher surface hardness named oxygen boost diffusion (OBD) treatment has been proposed by Dong et al.^8^. The OBD treatment embraces two steps: (1) thermal oxidation in air to form an oxygen reservoir, and (2) oxygen diffusion treatment of pre-oxidized samples in vacuum. Compared with thermal oxidation treatment, the OBD treatment can effectively improve surface hardness and wear resistance due to the generation of the thick oxygen diffusion zone (ODZ)^9^. However, being restricted by the reaction kinetics, the pursuit of a thicker ODZ generated by the method of increasing temperature or prolonging reaction time was not practical^10^. To overcome this issue, an increasing number of studies have focused on surface duplex treatment, especially on the grain refinement process followed by thermochemical treatment.

Ultrafine grains with a large number of grain boundaries can provide more diffusion paths effectively accelerating the diffusion process^11,12^. To generate ultrafine grains, mechanical surface treatments such as shot peening (SP), surface deep rolling (DP), surface mechanical attrition treatment (SMAT), and ultrasonic surface deep rolling (USDR) were widely used. Jia et al.^10^ applied deep rolling on pure titanium and then treated the strengthened material by the OBD method, through varying temperature and time. A thicker oxygen diffusion layer with higher hardness was generated, which was due to the higher non-equilibrium defects, grain boundaries, and more exposed crystal (101) surfaces. A similar result was also reported by performing shot-peening prior to plasma nitriding^13^. The duplex treatment produced a thicker nitrided layer and improved hardness down to a deeper area from the surface as compared to the nitrided-only specimens. Corrosion behavior and wear resistance can be also enhanced by such duplex treatment^14,15^. Meanwhile, the microhardness increased with the temperature of diffusion treatment that after surface mechanical nanoalloying treatment^16^. In addition to the improvement in surface properties, mechanical surface treatment followed by thermo-chemical technique could also impact the fatigue strength. Kikuchi et al.^17,18^ performed fine particle peening on stainless steel and subjected them to gas nitriding, resulting in an improvement of the fatigue strength. Gangaraj et al.^19^ investigated the effect of a combination of severe shot peening and nitriding on the fatigue behavior of low alloy steel. The result shows the fatigue limit had not been effectively improved, regardless of the order of severe shot peening and nitriding. Thus, such hybrid treatment was not always beneficial to fatigue improvement.

It has been confirmed that the ODZ can improve the surface hardness and wear resistance^7–9^. However, the effect of USDR-OBD treatment on the fatigue properties of titanium is still unclear. In this paper, the fatigue lives of pure titanium treated by USDR, OBD, and USDR-OBD combination method were explored.

## Methods

Experiments were performed on TA1 pure titanium with 30 mm in diameter. The chemical composition of the material is shown in Table S1. The as-received material was annealed in air at 680 °C for 2 h. Specimens for the fatigue test were cut from the annealed specimens. In order to characterize the surface properties of different treatments, the cylindrical specimens with 8 mm in diameter were also prepared.

**Figure 1:**
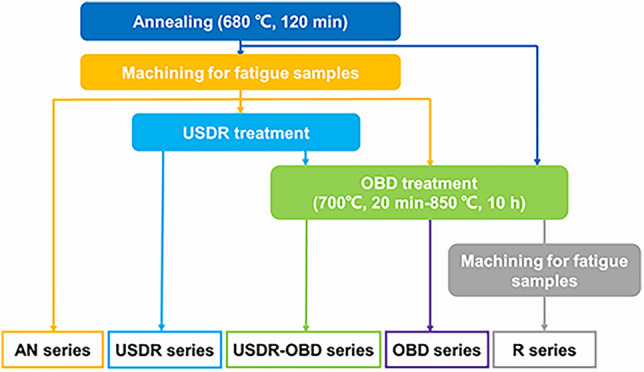
The flow chart of preparation process for 5 series samples.

Figure 1 illustrates the flow chart of specimen preparation. AN series represented annealed material without any strengthening treatment. The fatigue specimens of the AN series were then treated by the USDR process, called after the USDR series. The USDR treatment is an effective way to generate plastic deformation in the near-surface layer by means of its repeated squeeze and ultrasonic shock. The spherical rolling head used in this study was made from tungsten carbide of 10 mm in diameter. The vibration amplitude and the output frequency during the USDR process were 13 μm and 18.4 kHz, respectively. The rotation velocity of samples is 100 r/min, and the horizontally travel speed of the rolling ball is 10 mm/min. Thirty passes over the surface of each specimen were applied. USDR-OBD series specimens were treated by USDR treatment before OBD treatment. The OBD treatment was performed in two steps. The first one was pre-oxidation in the air at 700 °C for 20 min, which can provide enough oxygen potential as a reservoir for the diffusion process^10^, then the samples were taken out of the furnace for air cooling. During the second step, samples were further treated in vacuum (~ 1.3 × 10^–3^ Pa) with the diffusion temperature of 850 °C for 10 h. For comparison, the specimens only treated by OBD, i.e., OBD series, were also prepared. Moreover, to investigate the role of ODZ on fatigue behavior, the annealed materials were treated with the same heating process as OBD treatment (700 °C for 20 min in air and 850 °C for 10 h in vacuum), then were machined for fatigue specimens (removing ODZ), named as R series.

The surface microstructures of the treated specimens were observed by optical microscopy. The phase constituents of specimens were detected by X-ray diffraction analysis via Cu-K α radiation. The test parameters are as follows: the diffraction angle is 10–80°, and the scanning step was 0.02° for counting times of 5 s at each step. Vickers micro-hardness measurements were applied on the cross-sections of different samples with 0.49 g force and 15 s holding time. And the indentation distance was 10 μm to minimize the effect of the adjacent indentations on the hardness. Residual stress was measured layer by layer on an X-ray diffractometer using the sin^2^ψ method. The operation condition was set to 40 kV and 50 mA, X-ray wavelength was 0.154056 nm, the diffraction angle was 142°, diffraction plane was 213, the elastic constant was 11.888 × 10^–6^ and beam diameter was 4 mm. Electrolytic polishing was used to remove the material along with the depth. The electrolyte consisted of 5 vol% perchloric acid and 95 vol% absolute ethanol.

The strain-controlled tension–compression fatigue tests at the strain rate of 0.4% per second were carried out by INSTRON 8801 testing machine. Constant amplitude loading was applied with a strain ratio of −1 at a range of strain amplitude between 0.4% and 0.8%. An extensometer with a gauge length of 10 mm was used to measure the strain. The geometry of fatigue specimens is illustrated in Fig. S1. The roughness of the fatigue specimens should be less than 0.2 μm. Fracture surfaces were observed by Apollo 300 scanning electron microscope.

## Results and discussion

### Microhardness

**Figure 2:**
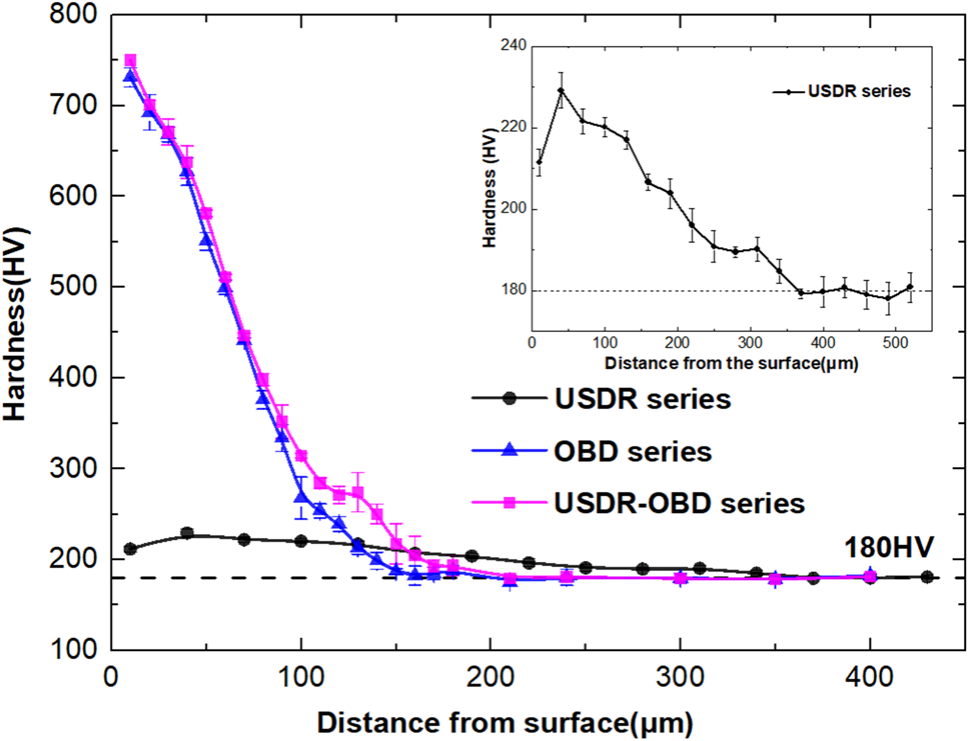
Microhardness along the distance from surface of USDR series, OBD series and USDR-OBD series.

Figure 2 shows the microhardness distribution on cross-sectional samples after various treatments. The average hardness of the AN series and R series are both 180 HV, which is shown as a benchmark line. It can be seen, compared with USDR series, the USDR-OBD series achieved a significant enhancement of hardness near the surface. The hardness gradually decreased with depth from ~ 750 HV to a stable value (~ 180 HV). At the same depth, the hardness in the USDR-OBD series was slightly greater than that in the OBD series, which indicated USDR treatment could somewhat enhance the oxygen diffusion process^10^.

### Microstructures

**Figure 3:**
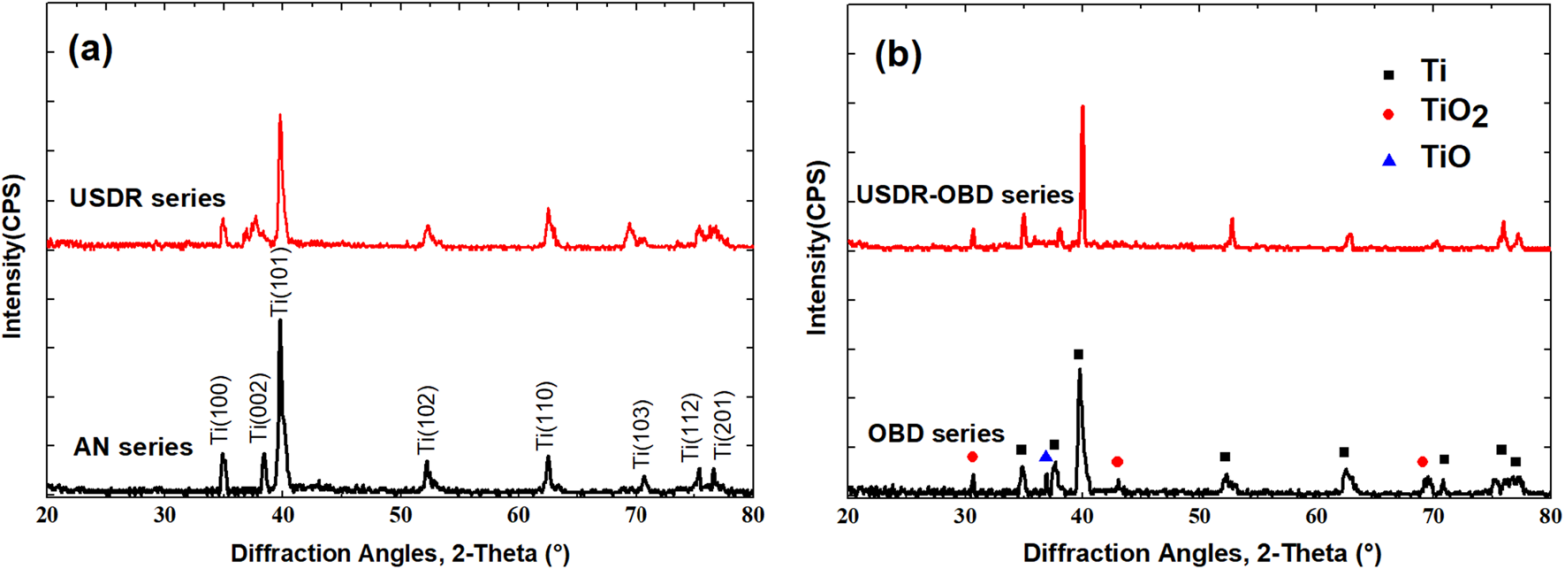
XRD patterns of (**a**) AN series, USDR series and (**b**) OBD series, USDR-OBD series.

Figure 3 shows the XRD patterns of samples treated by the USDR and OBD process. Compared with the AN series, the diffraction peaks of USDR treated Ti are broader and weaker, which was the result of refined grains and defects induced by the USDR process. After OBD treatment, an oxide layer was formed on the surface. It can be seen that some rutile peaks disappeared in the USDR-OBD series compared to the OBD series. This result indicated that USDR-OBD series samples had more oxygen atoms in the oxide layer dissolved and diffused into the Ti substrate than OBD series samples, which could be due to more grain boundaries and defects in USDR treated samples promoting the diffusion process.

**Figure 4:**
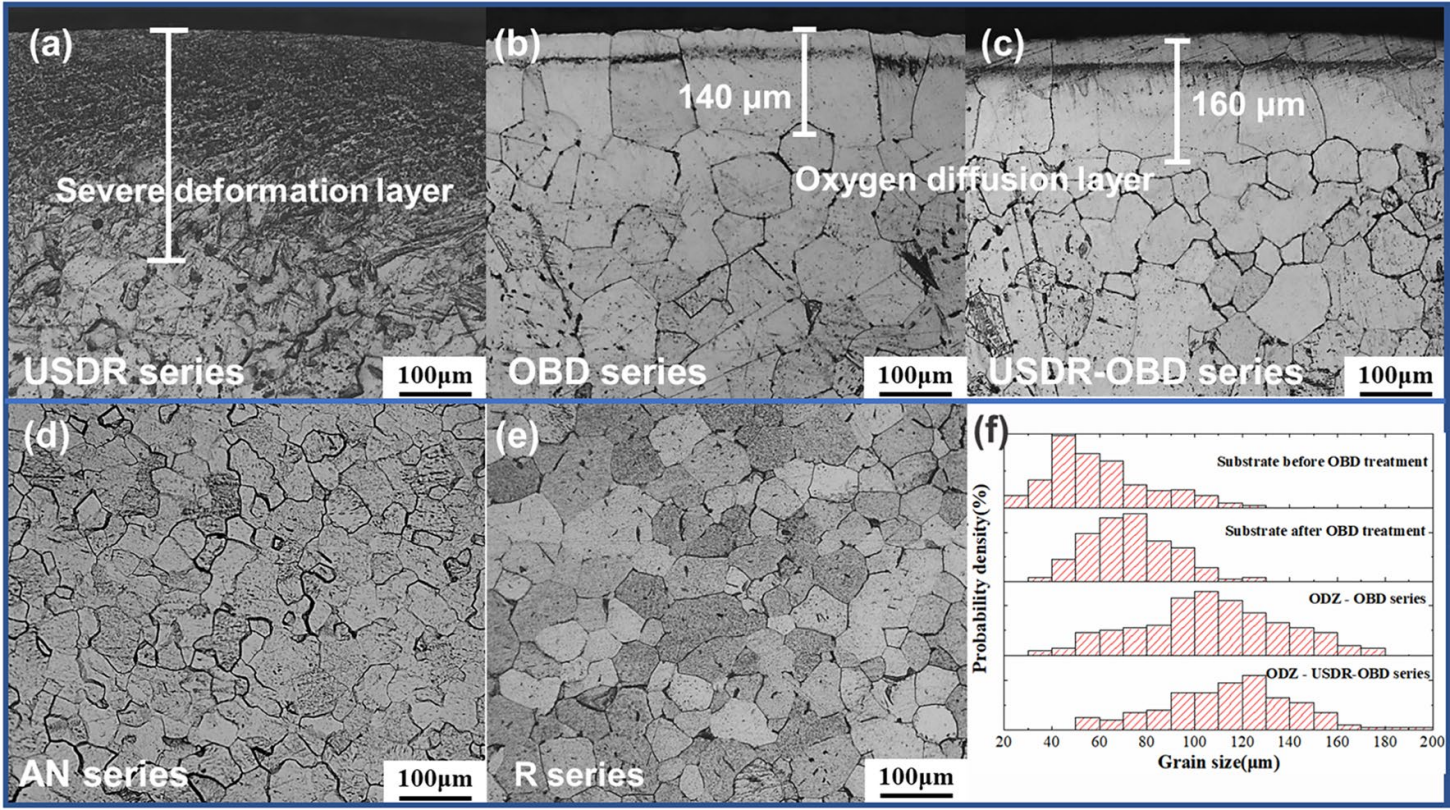
Microstructure from the cross section of (**a**) USDR series, (**b**) OBD series and (**c**) USDR-OBD series; microstructure from the substrate of (**d**) AN series, (**e**) R series; and (**f**) distribution of grain size in substrate and ODZ of different series.

Microstructures from the cross-section of the USDR series, OBD series, USDR-OBD series were shown in Fig. 4. The USDR treatment generated approximately 210 μm refinement layer from the surface. Within the severe deformation region, the grain size and grain boundaries were hardly distinguished by optical microscopy. According to the result of microhardness, the OBD and USDR-OBD treatment produced ODZ of about 140 μm and 160 μm in depth, respectively, which are shown in Fig. 4b,c. Meanwhile, according to Fig. 4d,e, the grain size of the substrate Ti changes significantly after the OBD process.

Five areas were randomly selected in the oxygen diffusion zone of the OBD series and USDR-OBD series to analyze the grain size of the ODZ. Meanwhile, to measure the effect of the high temperature during OBD treatment on grain growth in the substrate area, five random parts were also selected from the internal of samples before OBD treatment (AN series) and after OBD treatment (R series, OBD series, and USDR-OBD series). The grain sizes are shown in Fig. 4f. Statistically, the average grain size in the AN series was around 61 μm. After the OBD process, the substrate’s grain size grew to ~ 75 μm for both OBD and USDR-OBD series, while the grains in the ODZ of OBD series and USDR-OBD series grew to ~ 105 μm and ~ 114 μm, respectively. The grain growth in the substrate was the result of high temperature during the OBD process, while the significant grain coarsening in the ODZ was due to the lattice distortion caused by oxygen penetration, resulting in an increase in lattice parameters^20^. Thus, compared with the OBD series, the samples from the USDR-OBD series not only have the thicker oxygen diffusion zone but also show more significant grain coarsening. Meanwhile, it can be clearly seen that there was a layer of smaller grains under the ODZ in the USDR-OBD series, which was grown from fine grains at high temperatures. Thus, they didn’t appear in the OBD series. The average size of these small grains was around 56 μm, which was smaller than the substrate grain size of the USDR-OBD series.

### Surface roughness

The surface roughness had decreased from Ra 0.117 μm to Ra 0.050 μm after USDR treatment. This can be attributed to the continuous rolling of the rolling ball, which plays the same role as polishing. The roughness of the OBD and USDR-OBD treated samples was increased to Ra 0.129 μm and Ra 0.150 μm, respectively. This was due to the formation of pores at the top of the oxide layer^10^. Even though the surface roughness increased after OBD treatment, they still met the fatigue sample requirement.

### Residual stress

**Figure 5:**
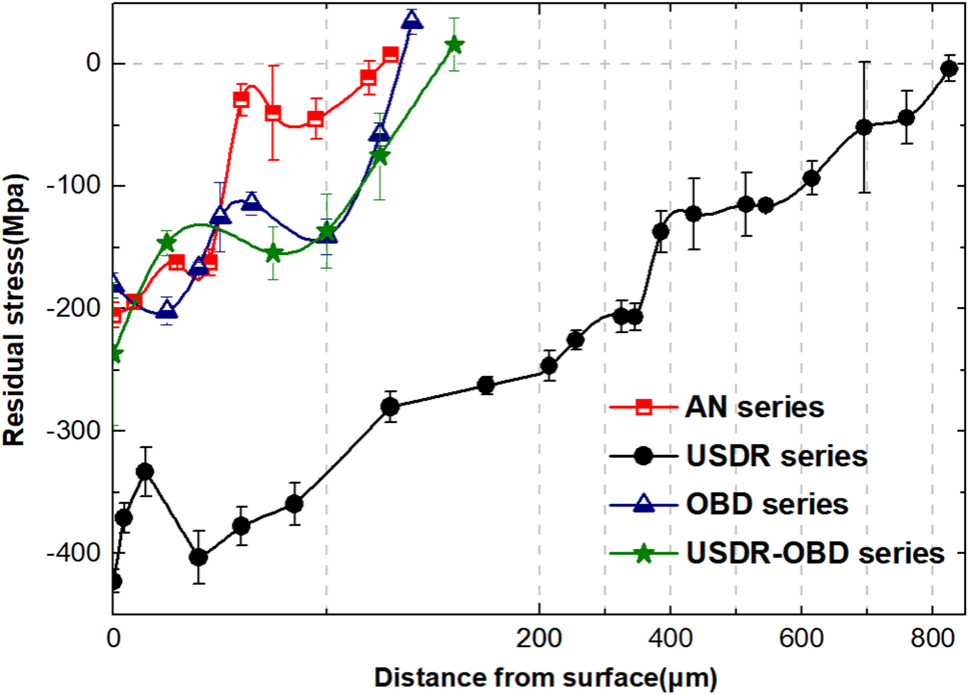
Residual stress distribution of AN series, USDR series, OBD series and USDR-OBD series.

Figure 5 shows the variation of the residual stress with depth from the surface for differently treated samples. At the near-surface region, compressive residual stress was generated under every treated condition. For the USDR series, ultrasonic rolling induced residual stress on the surface and subsurface through lattice distortion in plastic deformation. Compared to the USDR series, the subsequent OBD process decreased both the magnitude and depth of compressive residual stress, which was due to the residual stress release during high temperature. Moreover, one can note that the residual stress distribution in the OBD and USDR-OBD series had the same upward trend. The depth of compressive residual stress in both series was basically the same as the depth of the oxygen diffusion zone (ODZ), which could indicate that the residual stress in the OBD and USDR-OBD series was from the oxygen diffusion.

### Fatigue tests

**Figure 6:**
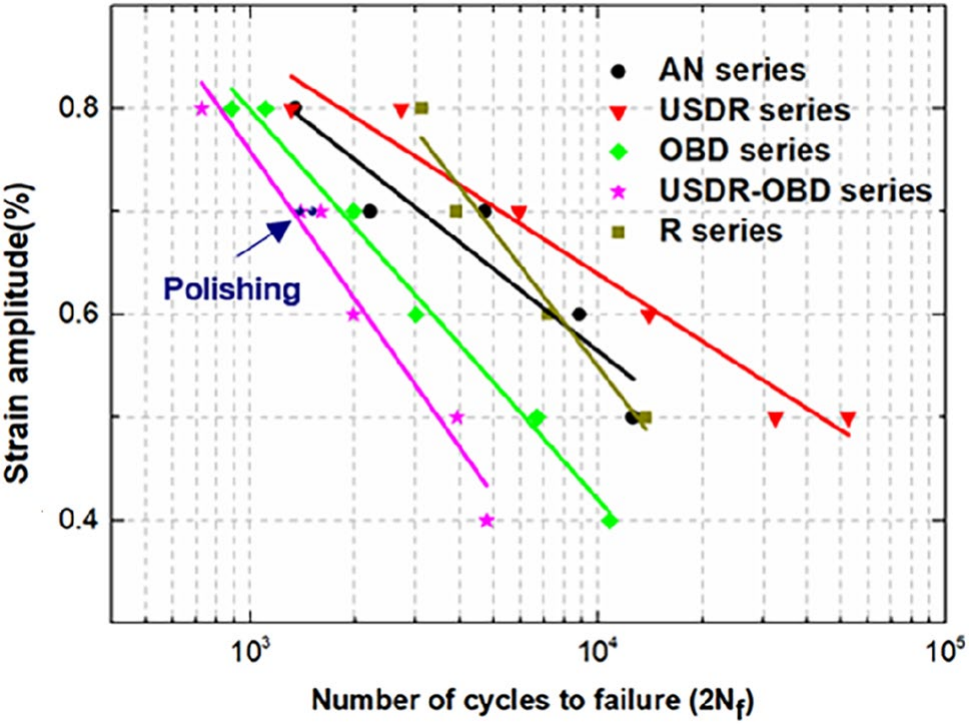
The strain-fatigue life curves of AN, USDR, OBD, USDR-OBD and R series.

**Figure 7:**
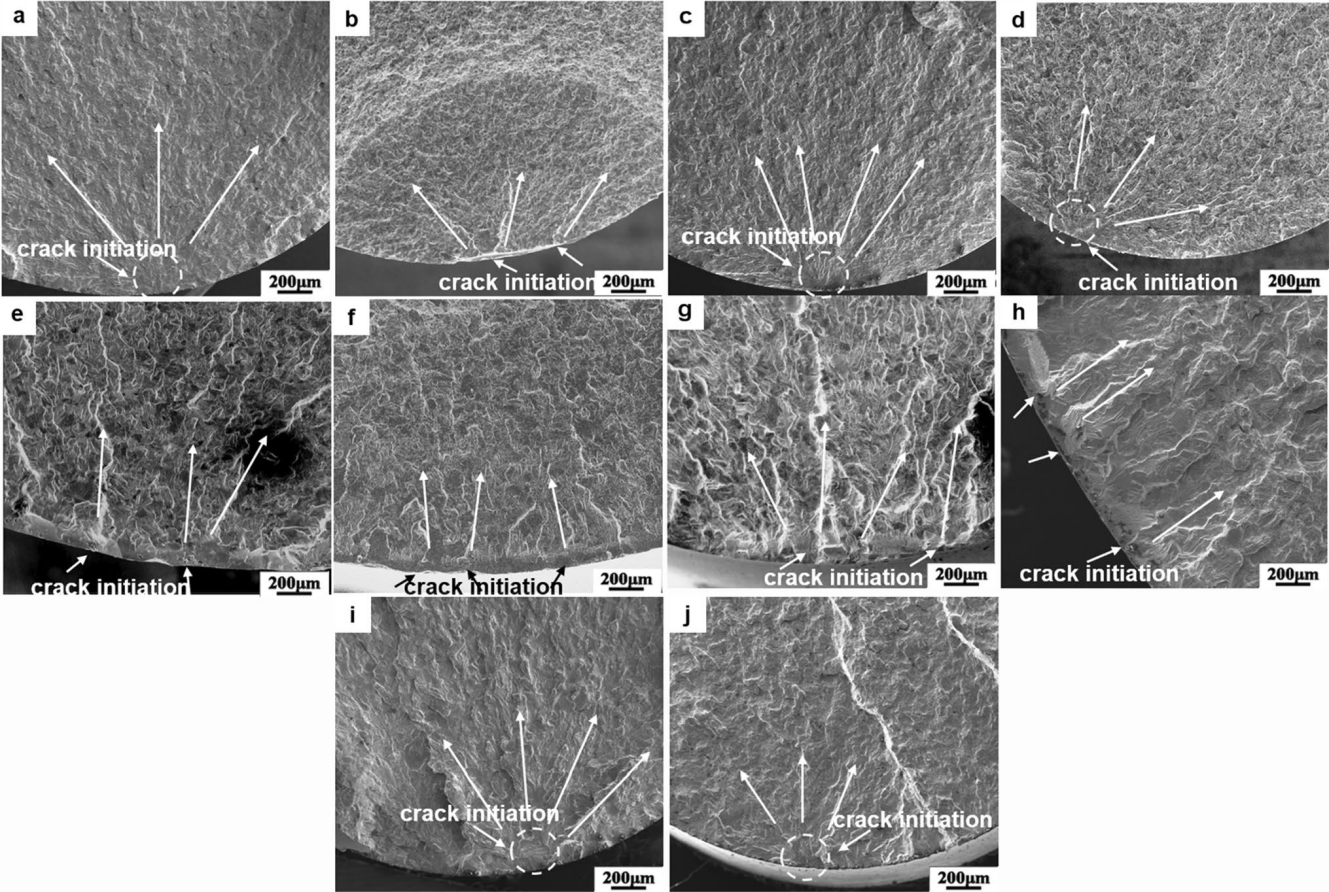
Fracture morphologies of different treated samples: (**a**,**b**) AN series at strain amplitude of 0.5%, 0.8%, respectively; (**c**,**d**) USDR series at strain amplitude of 0.5%, 0.8%, respectively; (**e**,**f**) USDR-OBD series at strain amplitude of 0.4%, 0.8%, respectively; (**g**,**h**) OBD series at strain amplitude of 0.4%, 0.8%, respectively; (**i**,**j**) R series at strain amplitude of 0.5%, 0.8%, respectively.

In order to investigate the effect of USDR and OBD treatment on the fatigue properties of pure titanium, uniaxial fatigue tests were conducted. The fracture surfaces of all treated samples were observed by SEM to figure out the effect of different surface treatments on fatigue crack initiation and propagation. The fatigue life curves of pure titanium by various treatments at different strain amplitudes can be seen in Fig. 6. Herein, the fatigue results of the AN series were set as a benchmark. After the USDR treatment, the life cycles of the USDR series increased 12.4% and 315.3% at 0.8% and 0.5% strain amplitude, respectively, which illustrates that USDR treatment effectively improved the fatigue life of pure titanium, especially in low strain amplitude. As shown in Fig. 7a–d, the fatigue crack sources were located at the surface for both the AN and USDR series. However, the USDR series presented a single fatigue crack initiation site at strain amplitudes of 0.8%, while the AN series showed the characteristics of several fatigue crack sources at high strain amplitude. That shows that the refined grains and the high compressive residual stress after the USDR process inhibited the fatigue crack initiation, contributing to the fatigue life improvement in USDR series^17,18^.

Furthermore, the fatigue life of the samples after OBD treatment, i.e., OBD and USDR-OBD series, was significantly decreased at the used strain amplitudes. Figure 7e,f show the fatigue fracture morphologies of the USDR-OBD series, in which several cracks were presented on the surface and propagated perpendicular to the specimen surface, indicating that the cracks were initiated suddenly at the relatively large size and grew into the bulk. The same tendency was found in the OBD series (Fig. 7g,h). That observation indicated that the ODZ didn’t delay the premature crack initiation at the surface, even it had higher hardness. The brittle ODZ would induce crack initiation faster. Similar results were reported in other studies^8,21^. As shown in Fig. 6, the fatigue life of the USDR-OBD series was lower than that of the OBD series, which could be related to the thicker brittle ODZ in the USDR-OBD series, resulting in earlier crack initiation.

During the OBD treatment, the samples had to keep a high temperature for a long time. As mentioned in Fig. 4d–f, there was an obvious growth between the grain sizes of the substrate before and after OBD treatment. Thus, the possible reasons could be speculated to be related to the size and structure changes in substrate grains. In comparison with the AN series, the fatigue life of the referenced samples, i.e., the R series, was basically higher, which indicated the grain growth beneath the surface of the cylinder contributed to the increasing fatigue life. Moreover, the fatigue crack initiation characteristics in the R series, as shown in Fig. 7i,j, were similar to those in the AN series. Meanwhile, the surface compressive residual stress caused by OBD or hybrid treatment was basically the same as the AN series, thus the residual stress played a less important role in life reduction. As most fatigue cracks initiate from the surface, the effect of surface roughness on fatigue life must be investigated. After OBD treatment, a residual oxide film still existed on the samples, so the surface roughness was slightly larger than that of the untreated series. In order to explore the effect of surface roughness on the fatigue life, the USDR-OBD treated samples were selected for polishing to remove the oxide film, and the surface roughness was controlled to the same as that of the AN series (~ Ra 0.110 μm). The fatigue life of the polished sample, under 0.7% strain amplitude, was basically the same as that of the USDR-OBD treated samples. Therefore, surface roughness was not the main reason for fatigue life reduction of USDR-OBD treated titanium. Meanwhile, the R series had a higher fatigue life than the OBD series. As the structural difference between the two series was dependent mainly on the presence of brittle ODZ, the higher fatigue life of the R series was mainly due to the removal of the brittle ODZ. The fatigue life of the USDR-OBD series was lower than that of the OBD series, that was, the thicker the ODZ was, the lower the fatigue life was. These results certainly confirmed the speculation that the brittle ODZ with coarse grains played an important role in the life reduction of OBD and USDR-OBD series specimens^8^. The tensile properties and indentation tests on ODZ were described from Fig. S2 to Fig. S3.

**Figure 8:**
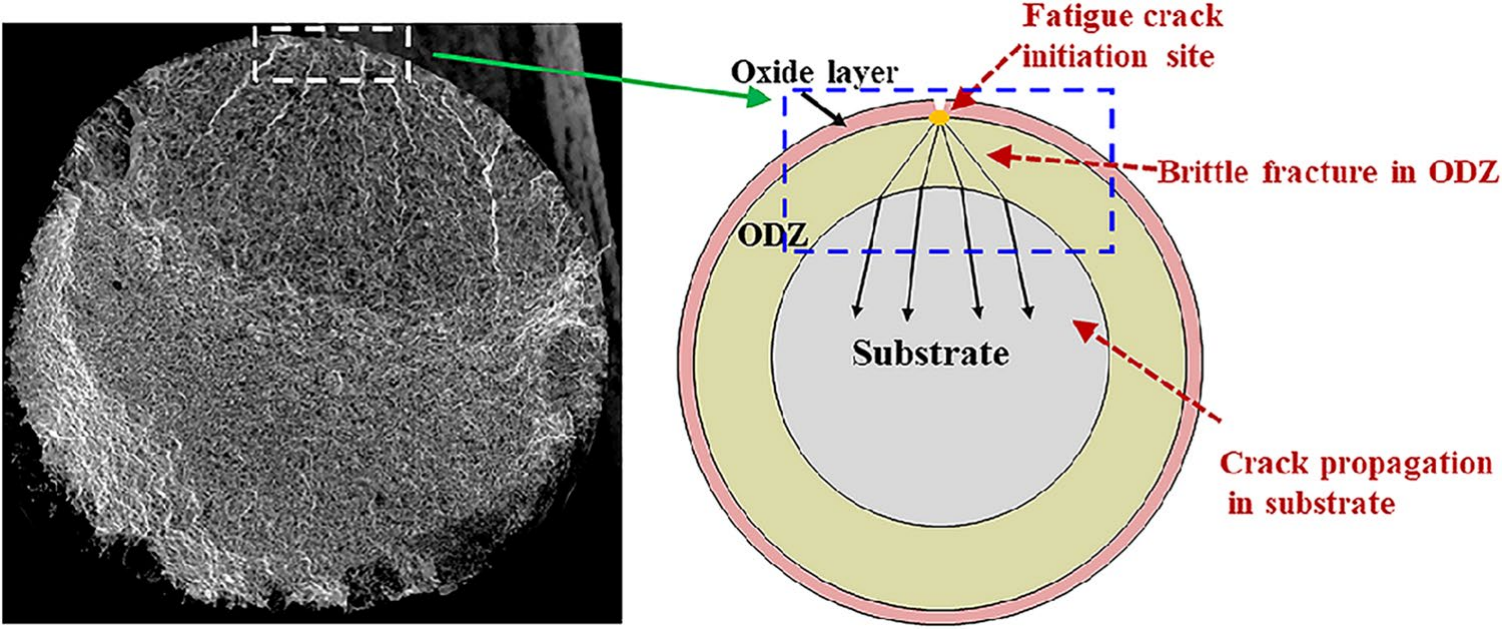
Schematic showing fatigue fracture process in USDR-OBD series.

As described previously, the fatigue crack originated from the surface of the USDR-OBD series. These premature initiation sites, generating at the interface of the residual oxide layer and the ODZ, could be related to their misfit dislocation^22^. The misfit dislocation resulted in stress concentration which became preferred nucleation sites under fatigue loading. After that, the microcracks formed and grew into the ODZ. The appearance of the fatigue crack became very shallow in the beginning and then grew into the substrate rapidly. The next step was the cracks propagated in the substrate before the final failure. Schematically, the fatigue fracture model for the USDR-OBD series is shown in Fig. 8.

In this paper, the combination of USDR-OBD treatment generated a harder surface layer, while the fatigue properties of pure titanium were decreased. As fatigue failure is one of the main causes of material fracture in industrial applications, it is particularly important to improve the fatigue properties of pure titanium. In order to obtain excellent surface properties as well as better fatigue properties, it is reasonable to decrease the grain size of ODZ by reducing the diffusion temperature. Tong et al.^23^ proved that nitriding at low temperature can effectively produce a high hardness surface layer by means of surface nanocrystallization treatment. Other studies also proved that it is feasible to improve surface properties by the combination of surface mechanical treatment and low-temperature diffusion treatment^24,25^. Thus, the effect of USDR plus OBD at lower diffusion temperature on fatigue properties should be further investigated.

## Conclusion

In order to form a thick and hard ODZ, USDR was performed prior to OBD treatment. Uniaxial fatigue tests were conducted to investigate the effect of the duplex treatment on fatigue behavior and fracture mechanism. Several conclusions can be drawn as follows:

1. The USDR-OBD series had a better hardening effect than the OBD series, indicating severe deformation layer enhanced OBD process by providing more diffusion paths.
2. USDR treatment improved the fatigue life of pure titanium, which was attributed to the high compressive residual stress and refined grains in the near-surface region.
3. Despite the high surface hardness, the compressive residual stress introduced by USDR treatment is released after OBD treatment, and the existence of the brittle oxygen diffusion layer leaded to premature initiation and rapid propagation of fatigue cracks, decreasing the fatigue life of pure titanium in USDR-OBD series.
4. In the USDR-OBD series, fatigue crack originated at the surface prematurely. Then brittle fracture occurred in the oxygen diffusion zone rapidly. Finally, the crack grew into the substrate before fracture.

## Supplementary Information

Supplementary Information.
