# Supplementary material for: Effect of ultrasonic surface deep rolling combined with oxygen boost diffusion treatment on fatigue properties of pure titanium
Source: Sci Rep. 2021 Sep 8;11:17840. doi: 10.1038/s41598-021-97255-x (PMC8426462; doi:10.1038/s41598-021-97255-x)
Supplement: Supplementary file 1 — Supplementary Information. [file 41598_2021_97255_MOESM1_ESM.pdf]

Supplementary information

Effect of ultrasonic surface deep rolling combined with oxygen boost diffusion treatment on fatigue properties of pure titanium

Xue-Fei Teng [1], Yun-Fei Jia [1*], Cong-Yang Gong [1,2], Cheng-Cheng Zhang [2],
Xian-Cheng Zhang [1*], Shan-Tung Tu [1]

1 Key Laboratory of Pressure Systems and Safety, Ministry of Education, School of Mechanical and Power Engineering, East China University of Science and Technology, Shanghai 200237, China

2 AECC Commercial Aircraft Engine Co. LTD, Shanghai Engineering Research Center for Commercial Aircraft Engine, Shanghai 201108, China

*Corresponding author: *E-mail address*: yfjia@ecust.edu.cn (Yun-Fei Jia)
xczhang@ecust.edu.cn (Xian-Cheng Zhang)

Supplementary Materials and Methods

Alloying Elements	Fe	C	N	H	O	Ti
Wt (%)	<0.083	<0.007	<0.02	<0.002	<0.08	Balance

Table S1. Chemical composition of TA1

Table S1 lists the chemical compositions of the pure titanium, which was provided by Baoji BaoTi metal products Co., Ltd.

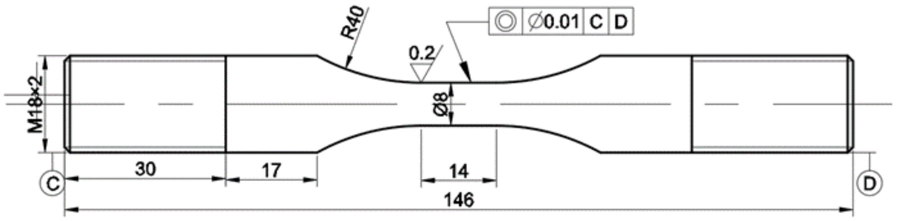

Fig. S1. Specimen for low- cycle fatigue test (unit: mm)

Figure S1 shows the geometry of fatigue specimens.

Supplementary Discussions

Tensile properties

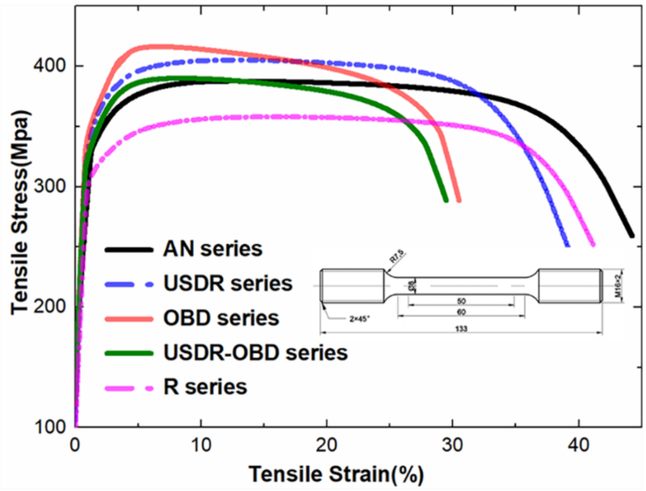

Fig. S2. The tensile curves of AN series, USDR series, OBD series, USDR-OBD series and R series.

Fig. S2 shows the tensile curves of pure titanium under different treated conditions. The load rate of uniaxial strain-controlled tensile test was 1mm/min. Compared with AN series, the strength and

plasticity decreased in R series. The reason may be the grain growth during the heating process. Because the grain boundaries act as obstacles to dislocation motion, the increment in yield strength decrease with the grain growth[1]. Thus, the strength and plasticity were reduced in R series. One can note the elongation to failure decreases for all strengthened samples. For OBD and USDR-OBD series, the elongation decreased about 23.8% and 29.5%, respectively, indicating that the OBD treatment reduced plasticity of pure titanium. Moreover, the elongation of OBD series decreased much more when comparing with that of R series. The major difference between OBD and R series is the existence of brittle ODZ in OBD series, which results in the decreased plasticity.

Nanoindentation results

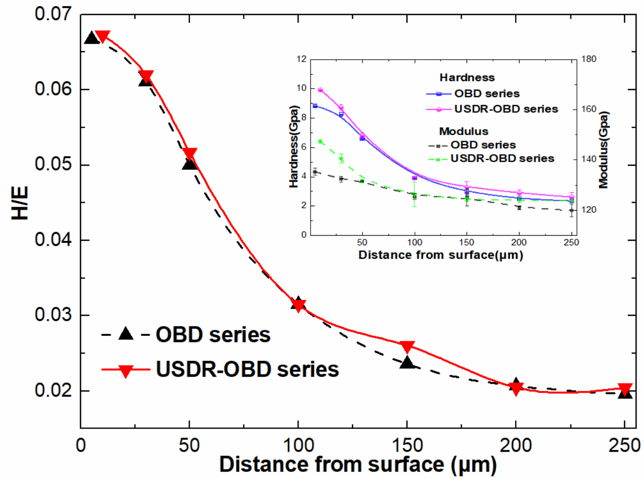

Fig. S3. The results of hardness, modulus, and H/E in OBD and USDR-OBD series

Fig. S3 Shows the results of hardness (H), modulus (E), and H/E in OBD and USDR-OBD series. To further investigate the properties of the ODZ, nanoindentation tests with a Berkovich indenter were conducted in USDR-OBD and OBD series. The elastic modulus, as well as hardness, decreased with the depth from the surface. Studies showed the interface stress was one of key factors resulting in the failure of materials[2]. Thus, the difference in mechanical property between ODZ and substrate is crucial for failure. The parameter of H/E has been used for describing the interface match[3]. From Fig. S3, H/E reached highest at the surface, and then gradually decreased with depth. Though, a big difference in H/E exists between the surface and the substrate, there was no sudden drop at the interface, indicating the ODZ had a good match to the substrate.

References

1. Huskms, E. L., Cao, B. &Ramesh K. T. Strengthening mechanisms in an AI-Mg alloy. *Mater. Sci. Eng. A* **527**(6), 1292-1298 (2010).
2. Zalesak, J. *et al.* Cross-sectional structure-property relationship in a graded nanocrystalline Ti1-xAlxN thin film. *Acta Mater.* **102**, 212-219 (2016).
3. Cao, Y. Z. *et al.* Fracture mechanism of an Al/AlN/CrAlN gradient coating on nitrogen implanted magnesium alloy. *Surf. Coat. Technol.* **302**, 126-130 (2016).
